# Quality antenatal care protects against low birth weight in 42 poor counties of Western China

**DOI:** 10.1371/journal.pone.0210393

**Published:** 2019-01-16

**Authors:** Hong Zhou, Anqi Wang, Xiaona Huang, Sufang Guo, Yuning Yang, Kathryn Martin, Xiaobo Tian, Jonathan Josephs-Spaulding, Chuyang Ma, Robert W. Scherpbier, Yan Wang

**Affiliations:** 1 Department of Maternal and Child Health, School of Public Health, Peking University, Beijing, China; 2 Environmental and Spatial Epidemiology Research Center, National Human Genetic Resources Center, Beijing, China; 3 Department of Epidemiology, Johns Hopkins Bloomberg School of Public Health, Baltimore, Maryland, United States of America; 4 UNICEF China, Beijing, China; 5 UNICEF Regional Office for South Asia, Leknath Marg, Kathmandu, Nepal; 6 Institute for Experimental Medicine, Christian-Albrechts-University Kiel, Kiel, Germany; 7 Beijing Lu He International Academy, Beijing, China; London School of Hygiene and Tropical Medicine, UNITED KINGDOM

## Abstract

Limited data exist related to low birth weight (LBW) incidence and risk factors in Western China. This paper aims to assess LBW and its relationship with antenatal care (ANC) in the poor counties of Western China. A community-based study in rural Western China was conducted in 2011. A kernel distribution was used to estimate the adjusted LBW incidence, and associations between LBW and socio-demographic or maternal factors were examined using multilevel logistic regression. Among 8,964 participants, 65.7% were weighed at birth. Crude LBW incidence was 6.6% and the adjusted rate was 9.3%. The study revealed that risk factors of LBW are being female, raised within a minority group, and with a family income below the national poverty line. For maternal risk factors, LBW was positively associated with not attending at least five or eight ANC visits, not receiving any ANC during the first trimester, and not having access to assess certain ANC content (weight, blood pressure, blood test, urine test, B-scan ultrasound, and folic acid supplement). There is urgent need to promote quality ANC in poor and rural areas of Western China and to prioritize vulnerable women and children who will benefit from quality ANC.

## Introduction

In both developing and developed countries, low birth weight (LBW) (live birth weight <2,500 g) is the leading cause of neonatal death as well as the primary reason for childhood mortality and morbidity [1]. Globally, 15.5% of infants are born with a below average birth weight; most of these births occur within developing countries [1]. LBW was estimated to account for 17% of all infant deaths in the US in 2010 and 22% in China in 2013 [2, 3]. LBW in infants is typically attributed to premature or restricted intrauterine growth.[1] Due to the immaturity of organs and insufficient body fat, LBW infants are more susceptible to infections, are unable to maintain body temperature, and have difficulty in both feeding and gaining weight. Premature infants also have an increased risk of developing health problems such as respiratory pathologies (e.g. respiratory distress syndrome), neurologic disorders (e.g. intraventricular hemorrhage), gastrointestinal diseases (e.g. necrotizing enterocolitis). and other ailments that require specialized hospital care. Moreover, the adverse effects of LBW impact a child’s health during development in later years; this includes a higher risk of obesity, diabetes, hypertension, heart diseases and low bone density [4–7]. For these reasons, scholars and researchers alike have been devoted to studying the determinants of LBW, especially the obstetric and socio-economic factors which seem to play a major role in the onset of LBW. The overall objective of this study is to provide evidence for effective antenatal interventions that can prevent LBW, and thus lower child mortality in vulnerable populations in rural Western China.

In China, the incidence of LBW was 5.9% in 1998 and 6.1% in 2011, which is lower than the global average [8, 9]. However, inequalities remain persistent among various regions and population groups within China. A previous study reported regional differences in China of 9.4% (Southwest China) and 2.5% (Central China) [8]. This geographical inequality of LBW incidence is consistent with childhood mortality distributions and suggests that bolstering maternal care and neonatal health is needed in Western China; however, there is limited data in recent years on LBW in Western China [10].

Many studies in developing countries have provided evidence that the improvement of quality antenatal care (ANC) can significantly mitigate the incidence of LBW. Quality ANC includes accessibility to ANC, the number of ANC visits, and the content of ANC [11–14]. To improve residential health care, a nationwide basic public health service has been provided by the Chinese government since 2009. This services also financially supports free prenatal care for rural women. At the time of this study, the Ministry of Health of China has recommended at least five ANC visits for all pregnant women [15]. Given this recommendation, there is an urgent need to evaluate the quality of prenatal examinations in Western China and to determine if the improvement of prenatal care adequately mitigates the occurrence of LBW in children from that region.

In this study, we assess the LBW prevalence in resource-deficient areas in Western China and examine the association of ANC quality on LBW delivery by conducting a population-based survey in forty-two counties in rural Western China.

## Methods

### Study areas

Data were collected from a community-based, cross-sectional survey conducted in forty-two counties, with a total population of 11, 000, 000, in seven Western provinces of China: Gansu, Guizhou, Qinghai, Yunnan, Sichuan, Tibet, and Xinjiang. The annual income per capita of this rural population in forty-two counties (3,239 yuan) was below the national average (5,919 yuan) in 2010 [16]. The under-five mortality rate was as high as twenty-one per 1000 live births in these counties as compared with a national rural average of sixteen per 1000 live births in 2010. Moreover, the fifty-one per 100, 000 live births for maternal mortality rate in these counties was also higher than national rural average of thirty per 100,000 live births in 2010 [16]. A written informed consent was obtained from each participant before enrollment. The survey was conducted by Peking University School of Public Health and UNICEF working with local health authorities and was approved by Peking University Health Science Centre’s Ethics Committee, in accordance with relevant guidelines and regulations.

### Data collection

A multistage sampling technique was used in each of the surveyed counties. First, fifteen administrative villages were randomly selected based on the probability proportional to size (PPS) methodology in each of the counties. Second, two rural villages in each administrative village were selected randomly with the PPS method. Third, in two natural village, ten children under three years old were randomly selected from a list of names of the local children who were younger than three-years-old. However, as the population in Tibet is sparsely distributed, only two or three children were randomly selected in each rural Tibetan village. Before the fieldwork, all interviewers had been trained using the same comprehensive standard. Face-to-face interviews with mothers or caregivers were conducted in 2011 using a structured questionnaire.

The structured questionnaires were designed using UNICEF’s Multiple Indicator Cluster Survey’s (MICS) format and modified on the basis of a literature review, expert consultation, and a pilot study [17]. The questionnaire included data on socio-demographic status (education, ethnicity, household size, etc.) of the household, annual household net income, the utilization of ANC, and nutritional supplementation taken during the pregnancy. Additionally, the survey sought whether the weight of the newborn was acquired at birth, if it was, then the birth weight of the newborn was taken. The mother would be asked to report the condition of her last child (in the case of twins, the younger child) if a mother had multiple children.

### Variables

LBW was defined as birth weights lower than 2,500 grams [1]. The question adapted from the MICS questionnaire (“Was the baby weighed when it was born? If so, what is the birth weight?”) was asked to the caregivers to obtain the birth weight. Birth weight was accurate to at least one decimal point beyond a kilogram. Educational status was categorized as illiterate (never attended any type of schooling), primary school (1–6 years of total education), secondary school (7–12 years) and college or above level (>12 years). Ethnicity was categorized as either Han or other minority groups (other ethnicity except Han, including Yi, Hui, Miao, etc.)[18]. Income was ranked according to the annual per capita net income for each household[19]. The annual household net income is equal to the value of household income minus the household expenditure in the past year before the survey. Household income included salary, family production value, (produce sold, animals slaughtered, output of milk, etc.) and the income from transfers and estate (gifts, donations, and other forms of transfer from community members). Household expenditure included spending on products (agricultural productive expenditure, livestock expenditure, etc.), living expenses (food, clothes, and commodities, residence, etc.), and expenditure for transfers and estate or tax. Aggregated annual household net income was divided by household size to determine per capita net income. Income was divided into two categories: below or above the China rural poverty line in 2011 (2,300 RMB)[20]. In addition, the number of siblings (at least 1 or 0) and place of delivery (county hospital, township hospital, or at home) were included in the analysis.

Based on international guidelines and national basic public health service guidelines in China [21–23], indicators for assessing ANC coverage (antenatal visit at least once, antenatal visits five or more times, antenatal visit at first trimester) and content of ANC (maternal weight, blood pressure, blood test, urine test, fundal height and B-scan ultrasound) were collected and included in the analysis for all children whose caregivers were mothers. Furthermore, indicators for assessing nutritional supplementation for mothers (including iron, calcium, folic acid and vitamins) were also analyzed for children if the caregivers were their mothers.

### Statistical analysis

Collected data was entered into a computer database and cleaned before analysis by using Epidata3.0 software package. STATA 15.0 for Windows statistical software package was used to carry out statistical analysis. Birth weights less than 250 or more than 5500 grams were classified as “missing”. The χ2 -test was used to examine the proportional differences between infants weighted and not weighted at birth. The significance level was set at p <0.05, two-tailed. To account for the abundance of infants at 2,500g (the cut-off point of LBW), the adaptive kernel smoothed cumulative distribution was fitted over the frequency distribution for birth weights and was used to estimate the adjusted LBW incidence [24, 25]. Socio-economic determinants and maternal factors for crude LBW without and with adjustment were all examined by hierarchical generalized logistic modeling (multilevel modeling) to account for clustering within the county level (ICC in empty model = 0.024). For maternal factors, the adjusted odds ratios (OR) with a 95% confidence interval (CI) was calculated and adjusted for the child’s birth year, sex, ethnicity, and family income. For all the analyses, a comprehensive weight coefficient was adopted to correct the different sampling probability and age distribution of each county. The datasets generated and analyzed during the current study are not publicly available due to the terms of consent to which the participants agreed, but are available from the corresponding author on reasonable request.

## Results

In total, 8,964 children aged 0–35 months were surveyed (response rate = 8,964 /10,058 = 89%). After applying the sampling and age distribution weight, there was a total of 5,891 children (65.7%) who were weighed at their birth, while 3,073 children (34.3%) were not weighed, as shown in Table 1. Sex of children in the weighed group did not differ significantly from those not in the weighed group (p = 0.178). However, children in the non-weighed group were more likely to have had at least one sibling (p<0.001), come from a minority group (p<0.001), live within a household with a low annual income (p<0.001), and have been delivered at home (p<0.001), in comparison to the weighed group.

**Fig 1:**
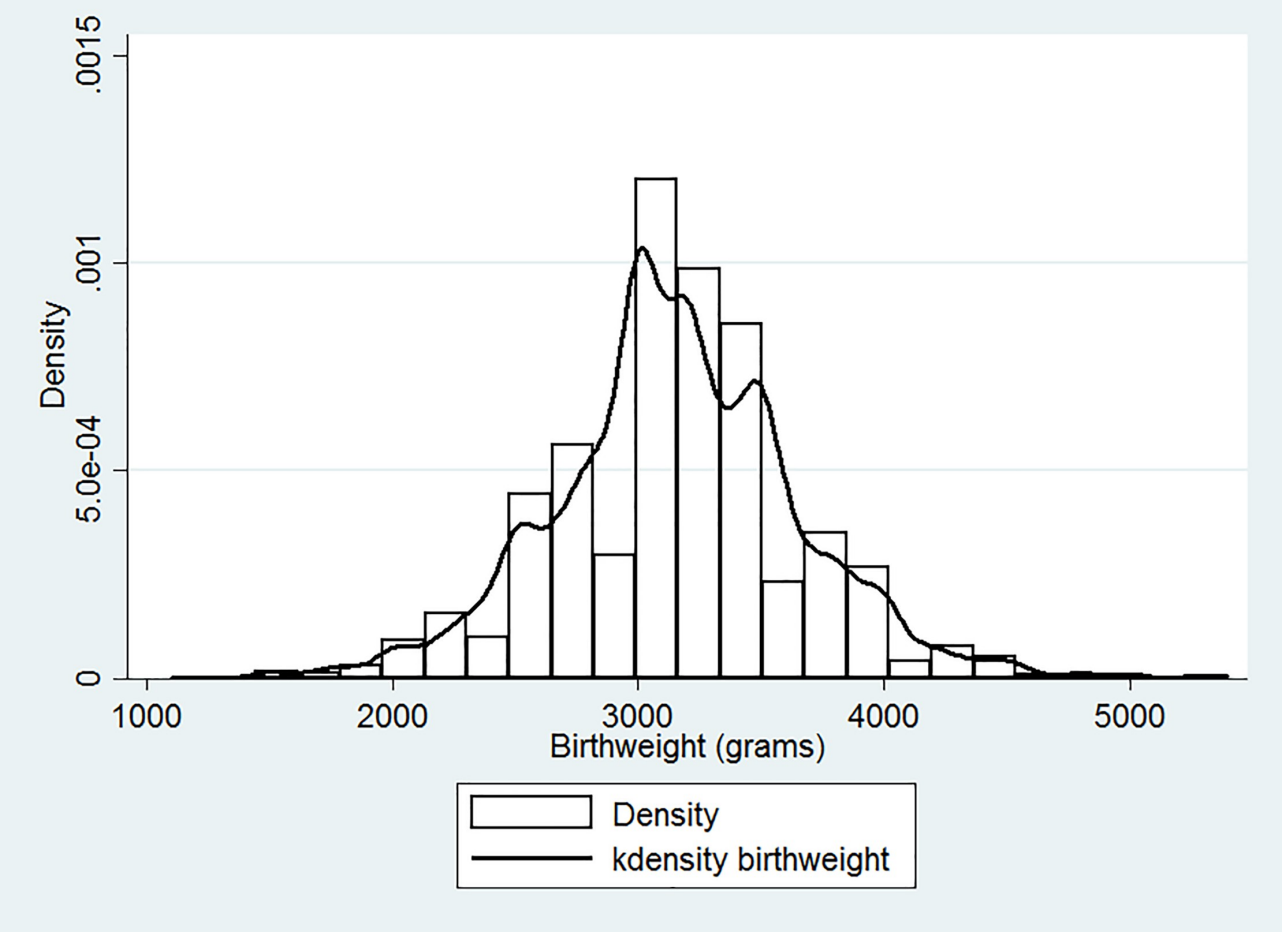
Histogram and Kernel distribution of birthweights.

Among the 5,891 children who were weighed at their birth, the crude LBW incidence of our sample population was 6.6% (387 cases), and 4.5% children were found to have received a rounded birth weight of exactly 2,500g as measured at their birth. The histogram and kernel density distribution of birthweight after adjusting for the abundance at 2,500g are shown as Fig 1. The density of multiples of 500 grams was substantially higher than others, especially for the bin of [2,500–2,600), which has the density of more than twice of the bin of [2,400–2,500). From the estimated density, we observed that approximately 9.3% of the newborns had LBW. As shown in Table 2, over time, the incidence of LBW decreased from 8.7% in 2008 to 6.1% in 2011. There was a significant statistical difference of LBW incidence between male (5.6%) and females (7.8%) (Table 2). Females had a higher risk of LBW than their male counterparts (adjusted OR (AOR) = 1.45, 95% CI = 1.11–1.89) after adjusting for the child’s birth year, ethnicity, number of siblings, delivery place, and family income. Similarly, the incidence of LBW among children from minority groups was significantly higher than that of Han children (AOR = 1.68, 95% CI = 1.26–2.24). Children from families below the poverty line had a higher probability of LBW than others (AOR = 1.27; 95%CI = 1.00–1.60). Children delivered at home and those without siblings also showed a higher incidence of LBW but did not show a statistical significant difference (p>0.05).

**Table 1:** Socio-demographic characteristics among children weighted or not at birth in 42 counties in Western China (N = 8964, 2011).

Characteristics	Weighted at birth	Not weighted at birth	Total	χ^2^	P
	n = 5891 (65.7%)	n = 3073 (34.3)	n = 8964		
	n (%)	n (%)	n (%)		
**Birth year of child**					
2008	357 (6.1)	266 (8.7)	623 (7.0)	135.049	<0.001
2009	1816 (30.8)	1236 (40.2)	3052 (34.0)		
2010	2045 (34.7)	957 (31.1)	3002 (33.5)		
2011	1672 (28.4)	615 (20.0)	2287 (25.5)		
**Sex of child**					
Male	3266 (55.7)	1653 (54.2)	4919 (55.2)	1.864	0.178
Female	2600 (44.3)	1399 (45.8)	3999 (44.8)		
**Number of siblings**					
0	3274 (58.5)	1089 (36.7)	4363 (51.0)	368.305	<0.001
1 at least	2319 (41.5)	1875 (63.3)	4194 (49.0)		
**Ethnicity**					
Han	2870 (48.9)	1001 (32.6)	3871 (43.3)	217.779	<0.001
Minorities	3005 (51.1)	2072 (67.4)	5077 (56.7)		
**Place of delivery**					
County hospital	4247 (73.8)	650 (22.0)	4897 (56.3)	4354.373	<0.001
Township hospital	1310 (22.8)	280 (9.5)	1590 (18.3)		
At home	197 (3.4)	2018 (68.5)	2215 (25.5)		
**Income**					
<2300	3801 (65.0)	2609 (85.5)	6410 (72.0)	421.242	<0.001
≥2300	2050 (35.0)	441 (14.5)	2491 (28.0)		

**Table 2:** Socio-demographic factors for low birth weight among children in 42 counties of Western China (N = 5891, 2011).

Characteristics	N	n	% (95%CI)	Crude OR^a^ (95% CI)	Adjusted OR^b^ (95% CI)
**Overall** **Birth year of child**	5891	387	6.6 (6.4–7.7)		
2008	357	31	8.7(5.4–12.0)	1	1
2009	1816	113	6.2(5.0–7.5)	0.70(0.43–1.12)	0.72(0.43–1.20)
2010	2045	140	6.8(5.6–8.1)	0.77(0.48–1.23)	0.81(0.49–1.32)
2011	1672	102	6.1(4.6–7.6)	0.68(0.41–1.13)	0.70(0.41–1.19)
**Sex of child**					
Male	3266	182	5.6(4.6–6.5)	1	1
Female	2600	202	7.8(6.6–9.0)	1.43(1.09–1.86)	1.45(1.11–1.89)
**Number of siblings**					
0	3274	231	7.1(6.0–8.1)	1.16(0.95–1.43)	1.22(0.93–1.61)
At least 1	2319	135	5.8(4.7–6.9)	1	1
**Ethnicity**					
Han	2870	143	5.0(4.0–6.0)	1	1
Minorities	3005	241	8.1(6.9–9.2)	1.67(1.27–2.19)	1.68(1.26–2.24)
**Place of delivery**					
County hospital	4247	271	6.4(5.5–7.2)	1	1
Township hospital	1310	93	7.1(5.3–8.9)	1.22(0.81–1.56)	1.16(0.83–1.61)
At home	197	17	8.6(4.0–12.8)	1.35(0.76–2.39)	1.31(0.73–2.36)
**Income**					
<2300	3801	262	6.9(5.0–7.9)	1.20(0.91–1.60)	1.27(1.00–1.60)
≥2300	2050	119	5.8(4.6–7.0)	1	1

^a^ Both the crude and adjusted OR were accounted for clustering within county.

^b^ Adjusted for all variables in the table.

Among the 5,891 interviewees of the children who were weighed at their birth, 4,311 (73.2%) of them were maternal guardians of the child. There was no significant statistical difference of LBW incidence between children with mothers as the interviewee (6.6%) and other caregivers as the interviewee (6.4%) (t = 0.39, p = 0.696). Table 3 examines the maternal factors for LBW among those children whose mother was an interviewee and summarizes maternal utilization of ANC during their most recent pregnancy. The proportion of mothers who had at least one antenatal visit was 88.9%, while the proportion of those who had at least five antenatal visits was 36.2%. Only 10.1% of mothers received gestational care at least eight times during antenatal visits. Moreover, the proportion of mothers who attended antenatal visits during the first trimester of pregnancy was 81.4%. Further analysis shows that children whose mothers who did not receive five ANC visits had a higher risk of LBW than those whose mothers had received it (AOR = 1.32, 95% CI = 1.01–1.73); likewise, the risk was similar if the mother did not attend eight antenatal visits versus attended eight visits (AOR = 1.54, 95% CI = 1.03–2.42). Although incidence of LBW among children whose mothers did not attend any antenatal visits or among those of mothers who had not received ANC at first trimester were high, there were no significant statistical differences when compared to the incidence among mothers who received the interventions (p>0.05), as shown in Table 3.

**Table 3:** Maternal factors for low birth weight among children in 42 counties of Western China (N = 4311, 2011).

Characteristics	N	n	%	Crude OR^a^	Adjusted OR^b^
	(%)		(95%CI)	(95% CI)	(95% CI)
**Overall **	4311(100)	286	6.6(6.1–7.7)		
**Mother’s education**					
Illiteracy	508(11.8)	35	6.9(4.2–9.5)	1.45(0.71–2.96)	1.55(0.73–3.33)
Primary	1254(29.1)	85	6.8(5.1–8.4)	1.45(0.74–2.84)	1.61(0.78–3.32)
Secondary	2409(55.9)	156	6.5(5.3–7.7)	1.21(0.64–2.34)	1.29(0.64–2.61)
College and above	136 (3.2)	11	8.1(2.7–13.0)	1	1
**Antenatal care coverage**					
At least once					
Yes	3604(88.9)	232	6.4(5.5–7.4)	1	1
No	448(11.1)	32	7.1(4.1–10.1)	1.05(0.68–1.62)	1.06(0.69–1.63)
**5 or more visits**					
Yes	1469(36.2)	70	4.8(3.6–6.0)	1	1
No	2584(63.8)	194	7.5(6.2–8.8)	1.34(1.02–1.76)	1.32(1.01–1.73)
**8 or more visits**					
Yes	435(10.1)	22	5.1(2.8–7.5)	1	1
No	3875(89.9)	264	6.8(5.8–7.8)	1.55(1.00–2.39)	1.58(1.03–2.42)
**Antenatal visit at first trimester**					
Yes	3304(81.4)	221	6.4 (5.4–7.4)	1	1
No	754(18.6)	60	8.0(5.4–10.4)	1.26(0.94–1.70)	1.36(1.00–1.85)
**Content of antenatal care**					
**Weight**					
Yes	3132(76.8)	194	6.2(5.2–7.2)	1	1
No	948(23.2)	82	8.6(6.3–11.0)	1.54(1.18–2.01)	1.49(1.14–1.95)
**Blood pressure**					
Yes	3322(81.5)	210	6.3(5.4–7.3)	1	1
No	754(18.5)	66	8.8(6.0–11.4)	1.50(1.13–1.99)	1.39(1.04–1.86)
**Blood test**					
Yes	2305(57.4)	133	5.8(4.7–6.8)	1	1
No	1711(42.6)	142	8.3(6.6–10.0)	1.50(1.17–1.91)	1.42(1.09–1.84)
**Urine test**					
Yes	2538(62.9)	159	6.3(5.1–7.4)	1	1
No	1500(37.1)	115	7.7(6.0–9.4)	1.38(1.08–1.77)	1.39(1.08–1.80)
**Fundal height**					
Yes	3071(75.9)	196	6.4(5.4–7.4)	1	1
No	977(241.)	79	8.1(5.9–10.3)	1.25(0.94–1.66)	1.18(0.88–1.59)
**B-scan ultrasound**					
Yes	3866(95.7)	248	6.4(5.5–7.4)	1	1
No	175(4.3)	20	11.4(6.7–17.5)	1.86(1.14–3.04)	1.71(1.00–2.93)
**Nutrients supplementation**					
**Iron**					
Yes	609(14.1)	42	6.9(4.7–9.0)	1	1
No	3695(85.9)	245	6.6(5.6–7.6)	0.88(0.61–1.26)	0.91(0.62–1.33)
**Calcium**					
Yes	202(47.1)	137	6.8(5.4–8.2)	1	1
No	2277(52.9)	149	6.5(5.4–7.7)	1.04(0.81–1.33)	1.00(0.77–1.31)
**Folic acid intake**					
Yes	1531(35.6)	84	5.5(4.1–6.8)	1	1
No	2773(64.4)	202	7.3(6.1–8.5)	1.33(1.02–1.72)	1.32(1.00–1.74)
**Vitamins**					
Yes	781 (18.2)	54	6.9(4.8–8.9)	1	1
No	3522(81.8)	231	6.6(5.5–7.5)	0.89(0.64–1.24)	0.96(0.68–1.38)

^a^ Both the crude and adjusted OR were accounted for clustering within county.

^b^ Adjusted for birth year, sex, ethnicity and income.

Additionally, Table 3 shows the proportion of each ANC component received by mothers during their most recent pregnancy. About 95.7% mothers received B-scan ultrasound, which was the most prevalent ANC service compared to others. Over two thirds of mothers had their blood pressure (81.5%), weight (76.8%), fundal height (75.9%), urine tests (62.9%), and blood tests (57.4%) measured. Analysis of the data showed that children whose mothers did not have their ANCs parameters meauserd, such as weight (AOR = 1.49, 95% CI = 1.14–1.95), blood pressure (AOR = 1.39, 95% CI 1.04–1.86), blood test (AOR = 1.42, 95% CI = 1.09–1.84), urine test (AOR = 1.39, 95% CI = 1.08–1.80) and B-scan ultrasound (AOR = 1.71, 95% CI = 1.00–2.93) had a higher risk of LBW in comparison to those whose mothers received them, respectively.

Lastly, Table 3 shows nutritional supplementation intake of women during pregnancy. All proportions of iron, calcium, folic acid, and vitamin intake were less than 50%. The highest was the proportion of calcium intake (47.1%), and the lowest was the proportion of iron intake (14.1%). Not taking antenatal supplements with folic acid was positively associated with LBW (AOR = 1.32, 95% CI = 1.00–1.74). Positive associations between other nutritional supplements and LBW prevalence were not found.

## Discussion

The study focused on LBW and ANC quality of impoverished rural Western China. The results revealed that about 9.3% of neonates born within the surveyed area had a LBW. Inadequate ANC visits and lack of certain ANC services were shown to be associated with of LBW after adjusting for other factors in the multilevel model. However, the ANC coverage, accessibility, and treatment quality are still low in the current poverty-stricken rural areas of Western China.

According to the most recent assessment, 8.1% of children in South China had a LBW in 2017 [26]. This prevalence is lower than in most developing countries and even in some developed countries (e.g. 28% in India, 11% in Thailand, and 8% in US) [27]. As estimated in the study, the overall incidence of LBW for the poor in rural Western China was 9.3%, which was relatively higher in comparison with LBW incidence in Eastern China. As estimated in other studies using medical facility-based data, the overall incidence of LBW in mainland China was 6.1% in 2011 [8]; 4.4% in Shanxi Province in 2010; 4.0% in Beijing in 2010 [28]; and 1.2% in Wuxi, a developed city in Jiangsu Province, in 2008 [29].

ANC is crucial for detecting infant abnormalities during crucial windows of neonatal development. This is especially true for women living within resource-constrained areas. Early detection ensures timely diagnoses and therapeutic interventions which ultimately contribute to averting LBW and other general complications which may negatively impact both the mother and neonate alike. In the analysis, at least five or eight ANC visits during pregnancy were shown to have protective effects for the mitigation of LBW. The cut-off point of eight ANC visits had a stronger association with LBW (higher OR) than five visits, but only few women could reach this indicator. A study in Brazil has shown that at least seven visits were protective in reducing LBW incidence; in comparison there were no significant differences of LBW incidence between mothers who received 4 or more visits and 1–3 visits in Nepal [11, 12]. The newly released WHO guidelines have been revised to recommend that at least four to eight contacts between the mother and health care providers are necessary to reduce perinatal deaths and improve the pregnancy experience [22]. However, the recommendation provided by the China National Basic Public Health Service Regulation suggests only five ANC visits during pregnancy [21]. Further evidence is needed in determining the optimal number of ANC visits to mitigate LBW in China.

The sex ratio of our study population is skewed from the natural sex ratio at birth (male: female = 1.23); higher than the sex ratio at birth in China of the same period (1.16–1.17 in 2008–2011).[30] Given that such an imbalanced sex ratio could be a consequence of the male preference and sex-selective abortion, and associated with other socio-economic and ANC factors, we used the chi-square test to examine the association between sex of the child and and maternal education, family income, ultrasound uptake and number of ANC visits, respectively. The results showed that there is no significant association between sex and each of those factors (p>0.05). This indicates that though gender selection may exist in the study area, the selection is not relevant in relation to the factors in which we are interested. A possible explanation for the unbalanced sex ratio is that we only included the youngest child in each household, and many rural families tended to stop giving birth when they had a boy. Thus, the suspected confounding effect of sex should not be huge concern to the association between other socio-economic, ANC factors and LBW in our study population. In addition, in the multivariate logistic regression analysis, we adjusted for the SES factors, which would account for a confounding effect, if it existed.

Numerous observational studies have demonstrated that the adequacy of ANC contents (such as the number of ANC visits adjusted to gestational age, completeness of care, or quality of prenatal examinations) were associated with LBW and many other pregnancy outcomes [11, 14, 31]. The study examined the association of ANC checklist items as recommended by the WHO (maternal weight measurement, blood pressure measurement, blood testing, urine testing, fundal height measurement and ultrasound scan) in relation to LBW incidence [32]. Even though the results illustrated that nearly all items, with an exception of fundal height measurement, were positively related to the reduction of LBW risk, the overall coverages were far from achieving 100% (with an exception of 96% coverage of ultrasound scan). Moreover, the high coverage of ultrasound in contrast to the low coverage of other contents could suggest an overuse of this examination. Some clinics might only conduct an ultrasound during an ANC visit, and thus misdiagnose health problems that the ultrasound cannot detect. The findings revealed poor ANC accessibility in the surveyed area; even though policies dictate that prenatal care is to be offered free of charge to all women in rural areas, studies have shown that there is a large proportion of women in rural Western China who have failed to reap the broad benefits of this policy[33]. Results from this survey show that about 10% of women sampled in our survey did not receive any form of ANC during pregnancy. Additionally, this study found that two-thirds women received less than five ANC visits; even among the women who received ANC, many of them received incomplete interventions. Due to these findings, we recommend the improvement of ANC coverage as well as quality in the poor rural western areas of China. Further studies should focus on identifying the barriers for women to access quality ANC in these rural communities.

Previous studies have demonstrated that taking nutritional supplements during pregnancy can mitigate the risks of gestational complications and is advantageous to pregnancy outcomes [34–36]. However, the proportion of surveyed women receiving at least one type of nutritional supplement was only 15%-43%. WHO’s ANC guideline recommends daily iron (30-60mg) intake for all pregnant women and daily calcium (1.5–2.0g) supplementation for pregnant women with low dietary calcium intake to prevent adverse health outcomes, including LBW [22]. Unfortunately, there is still a significant gap between the current Chinese maternal care guidelines and WHO recommendations. So far, neither Chinese Dietary Guidelines or the National Basic Public Health Service Regulations have adapted their guidelines or regulations based on the most current and relevant content [21, 37]. Folic acid (0.4mg) is also recommended by WHO through the entire pregnancy to prevent LBW and other diseases [22]. Before 2016, folic acid was only recommended 3 months before and after pregnancy by China's guidelines to prevent birth defects [33]. Since 2016, the Chinese Dietary Guideline for Women and Child has revised the recommendation for folic acid, requiring daily intake of 0.4 mg for all pregnant women, which is consistent with the current WHO recommendations [37]. However, the Chinese prenatal care guidelines have not been adjusted to reflect the WHO’s recommendation of folic acid intake, therefore daily folic acid has not been widely adopted throughout China, especially in rural Western areas. Further evidence on nutritional supplements during pregnancy is urgently needed.

One of the strengths of this study is its application of the kernel density estimation to assess the incidence of LBW in rural Western China. Kernel density estimation is a nonparametric technique that estimates the probability density function of a continuous random variable. Birth weight was typically rounded up to multiples of 500g when reported by participants or recorded in rural clinics. In the data, the birth weight of children was self-reported by caregivers rather than obtained from medical records, with 4.5% of the weighed children were reported exactly 2,500g; a contributor to these data points could be the possible result of rounding up [38]^,^[39]. Charnigo et al. suggested that birth weight distributions may be composed of sub-populations with different properties [40]. Therefore, we used the kernel density estimation to give a “smoothing” distribution of children’s birth weight and to adjust for the skewed distribution due to an abundance of reporting for 2,500g. Another strength is that the population for this study contained children born at home and in the hospital. Using only hospital-based sampling would have been more accurate; however it would have missed pertinent birth weight data. Results from this study suggest that children born at home tend to have lower birth weight in comparison to those who are born in a hospital. The use of community-based data allows for the capture of key mother and child groups who need interventions the most: those who gave birth at home and those with poor ANC accessibility.

The study notes that there are several limitations that raise concern when acquiring an estimation of LBW incidence. First, the birth weight of numerous children in this study was obtained by collecting self-reported weight from caregivers rather than from professional medical records; the memory of caregivers may not always be accurate and may lead to some recall bias, which could lead to an over or under-estimated incidence. Second, there was a large proportion of unweighted children in the study. Nearly one third of the children in this survey were not weighed at birth and were excluded in the following analysis. When comparing the unweighted children to those weighted, the unweighted children were more likely to be delivered at home and have a lower socio-economic status, which may make them more vulnerable to have a LBW. Therefore, the missing birth weights of these unmeasured children may cause an underestimation of the true magnitude of the LBW problem in the surveyed area.

Another issue that raised concern in this study is that not all known risk factors, such as maternal medical conditions and behavioral factors, were considered. Several studies have provided evidence that during pregnancy, the mother’s nutritional status; behaviors (e.g. alcohol, tobacco or drug using); maternal infections; and complications, such as pre-eclampsia or diabetes, may affect the growth and development of the fetus as well as the duration of the pregnancy [7, 41–43]. Moreover, it is a limitation that the study does not cover pregnancy complications and gestational age in the dataset. For those mothers with gestational hypertension or diabetes, their pregnancy is often induced before full term and results in LBW. Additionally, we did not collect mother’s history of preterm birth or LBW. Mothers who had a preterm or LBW delivery previously have a larger probability of LBW and also could be more health-conscious during consequential pregnancies.

In addition, this study only measured the contents of ANC and number of visits that women received during pregnancy, which only represents the overall quality of ANC in the study area to a certain extent. In China, ANC is part of the basic public health services regulated by the central and local governments, and the quality of ANC is mostly influenced by the institutions providing it. Unfortunately, we did not measure the institutional quality of ANC. Further studies regarding ANC quality should focus on both the services women receive and the institutions that provide those services.

To conclude, based on our estimation, LBW is still a noteworthy public health problem in Western China. LBW is more common among female children, those born at home, rhose born within an ethnic minority, and those from a poor family. As the study suggests, adequate and quality ANC care is associated with LBW incidence. The study also revealed an insufficiency of ANC in rural Western China. LBW incidence is a crucial indicator of maternal and child health progress and is closely associated with childhood and adulthood mortality and morbidity. Currently, there are scarce data on LBW in China, especially in remote areas of the Western regions. This study and its results contribute to a broader understanding of this issue and can help improve future allocations of ANC services and strategic investments to vulnerable groups impacted by LBW.

### Ethical approval

All procedures performed in the studies involving human participants were in accordance with the ethical standards of the institutional and/or national research committee and with the 1964 Helsinki Declaration and its later amendments or comparable ethical standards.

### Informed consent

Informed consent was obtained from all individual participants included in the study.
